# Happy Little Benefactor: Prosocial Behaviors Promote Happiness in Young Children From Two Cultures

**DOI:** 10.3389/fpsyg.2020.01398

**Published:** 2020-06-30

**Authors:** Yue Song, Martine Louise Broekhuizen, Judith Semon Dubas

**Affiliations:** ^1^Developmental Psychology, Utrecht University, Utrecht, Netherlands; ^2^Child, Family, and Education Studies, Utrecht University, Utrecht, Netherlands

**Keywords:** altruism, prosocial behaviors, happiness, toddlers and preschoolers, warm glow

## Abstract

Evidence that young children display more happiness when sharing than receiving treats supports that humans, by nature, are prosocial. However, whether this “warm glow” is also found for other prosocial behaviors (instrumental helping and empathic helping) and/or in different cultures is still unclear. Dutch (studies 1 and 2) and Chinese (study 3) young children participated in a sharing task, followed by instrumental helping and empathic helping tasks in which they were praised (thanked) if they helped. Consistent results were found across three studies, showing that (1) participants displayed more happiness after giving than receiving treats; (2) toddlers displayed more happiness after instrumental helping than initially interacting with the experimenter; and (3) toddlers’ happiness remained the same after positive social feedback (i.e., being thanked). Taken together, these results indicate that independent of culture, both sharing and instrumental helping are emotionally rewarding, supporting an evolutionary origin of these behaviors.

## Introduction

Sacrificing one’s own resources to support others, even strangers, is an exceptional human ability that has puzzled researchers for many years. While essential for establishing large-scale social cooperation in early human groups (e.g., Darwin, 1871; Wilson, 1975; Ebstein et al., 2010) and maintaining large organizations in the modern world (Wilson, 1975; Fehr and Fischbacher, 2003), the question is what benefits are there, if any, for the individual who is sacrificing? While theories such as kin selection (Hamilton, 1964) or reciprocal altruism (Trivers, 1971) focus on the potential, long term rewards that sharing or cooperation may bring (Aknin et al., 2015), a growing body of studies shows that the prosocial behavior itself might also be emotionally beneficial for an individual, suggesting additional short-term rewards (e.g., Dunn et al., 2008; for a review, see Aknin et al., 2018). For adults, prosocial behaviors (e.g., spending money on others) have been found to lead to an increase in happiness in both western and non-western cultures and across diverse socio-economic contexts (e.g., Dunn et al., 2008; Aknin et al., 2009, 2013, 2015). Based on these findings, these emotional rewards have been proposed as an evolved psychological mechanism that sustains prosocial behaviors even when it costs individual resources (e.g., Dunn et al., 2008; Aknin et al., 2013).

This “positive feedback loop” (Aknin et al., 2018, p. 55) also has drawn great attention from researchers studying young children. While many studies have focused on how (positive) emotional arousal serves as a precursor to toddlers’ and young preschoolers’ prosocial behaviors (e.g., Hepach, 2017; Cowell et al., 2018; Miller, 2018), another line of research has focused on whether and in which social context prosocial behaviors themselves are emotionally rewarding. A large number of studies have shown that prosocial behaviors can alleviate children’s negative emotions. For instance, even 2 years old negative arousals were alleviated both when they helped a stranger themselves (i.e., helped to retrieve the object that was out of the experimenters’ reach), and when a third-party provided help (Hepach et al., 2016). In fact, children who exhibit more empathic concern for others, helped faster (for a review, see Eisenberg and Miller, 1987). Furthermore, some researchers even propose that some prosocial behaviors (e.g., comforting) are motivated by alleviating one’s own negative feelings (for a review, Paulus, 2014).

In comparison, fewer studies have focused on how prosocial behaviors contribute to an increase of positive feelings, rather than just alleviating negative feelings. Three studies directly explored whether prosocial behavior *leads to* an increase of happiness in young children, by rating and comparing their happiness before and after sharing. The first study, conducted in Vancouver, Canada, gave toddlers windfalls (i.e., 8 treats), and then asked them to share one of these treats with a toy monkey (i.e., “I do not see any more treats, will you give one to monkey?”), and also share from other resources (i.e., look, I found one more treat, will you give it to monkey?). Results showed that 22 months old were rated as happier when they shared treats rather than when they received treats, and happier under a costly sharing condition (i.e., sharing from their own resources), rather than under a non-costly sharing condition (i.e., sharing from other resources) (Aknin et al., 2012). This study demonstrates that the relation between sharing and happiness already exists when sharing first emerges, and the results suggest this immediate emotional reward of sharing is a proximate mechanism that facilitates prosocial behavior despite its costs (Aknin et al., 2012). Using the same experimental paradigm, these results were replicated in a second study conducted among 2–5 years old children living in a remote, small rural village on Tanna Island (Aknin et al., 2015). More recently the emotional benefits of sharing was found among 3 and 5 years old Chinese children but only when sharing was autonomous rather than obligated (Wu et al., 2017). Nevertheless, before firm conclusions about this mechanism can be drawn, more studies are needed to test this universal claim.

Therefore, the first aim of the current study was to replicate and extend these findings by examining whether sharing behavior itself can lead to an increase of happiness among young children (i.e., toddlers and young preschoolers) in different cultures. For this purpose, we employed the same experimental procedure and rating scales used in the original study (Aknin et al., 2012), and we tested this proposed mechanism in three samples (i.e., Dutch toddlers, Dutch preschoolers, and Chinese preschoolers). More specifically, the first study aimed to replicate previous findings in toddlers. In addition, even though the warm glow is potentially detectable in all cultures, it “may vary in degree of expression according to the cultural context” (Aknin et al., 2013, p. 636). Considering that upon reaching the preschool age, children begin to internalize cultural norms, and orientations (Schuhmacher and Kärtner, 2015), it is plausible that the cultural context could play a role in young preschoolers’ prosocial behaviors, and further affect the happiness shown after prosocial behavior at this age. Thus, we also examined the warm glow in 3 years old among Dutch (Study 2) and Chinese (Study 3) preschoolers, when the warm glow may begin to vary in different cultural contexts. If the emotional reward of generosity is a universal and robust psychological mechanism, both Dutch and Chinese toddlers and younger preschoolers should express more happiness after sharing rather than receiving treats, and also after costly sharing compared with non-costly sharing.

Next to replicating these findings, our second aim was to examine whether the number of resources available when sharing affects the degree of happiness shown. There are reasons to think that resource availability might influence the happiness gain after sharing for toddlers and young preschoolers. In order to share, children need to overcome their own desire for the material resources (Dunfield, 2014). However, young children just engage in, and are sometimes even reluctant to share (Ulber et al., 2015), indicating that it may be difficult to overcome these desires. Accordingly, it is plausible that the increase in happiness after sharing is larger when individuals have a larger compared to a smaller amount of resources. That is, the more resources available, the more likely it is that their own material needs are still fulfilled when they share one resource, leading to a higher increase in happiness compared to children with fewer resources. In addition, cognitively, even toddlers can already discriminate a small set of objects (the numbers “one” to “four”) from a larger set of objects (more than four) (Xu, 2003; Sarnecka and Gelman, 2004; Le Corre and Carey, 2007). Thus, the understanding of number may allow toddlers to recognize the cost involved in sharing based on the number of resources available. Finally, studies on sharing behavior showed that young preschoolers are more likely to share after receiving more resources (Posid et al., 2015). The current study further examines whether children’s happiness after sharing is affected by resource availability. In previous studies on the happiness after sharing, children received a relatively large number of resources (8 treats) (Aknin et al., 2012, 2015). The current study went a step further by comparing the increase in happiness in three resource conditions: 2, 4, and 8 treats. If the increase in happiness after sharing is larger in the large set condition than in small set conditions, then this would support the idea that children’s own material needs need to be met (i.e., having enough resources) as a prerequisite for the happiness after costly sharing at this age.

The third aim of this study was to examine whether the emotional rewards of sharing also apply to other prosocial behaviors. Studies on the emotional rewards of prosocial behaviors mainly focus on sharing (e.g., Aknin et al., 2012, 2015), but there are more types of prosocial behaviors, such as instrumental helping and empathic helping, that already emerge at toddlerhood (e.g., Dunfield et al., 2011; Svetlova et al., 2011; Brownell et al., 2013; Dunfield and Kuhlmeier, 2013). More importantly, these three prosocial behaviors (i.e., sharing, instrumental helping, and empathic helping) are distinct from each other (i.e., requested for by different cognitive abilities, and driven by different motivations, for a review, see Padilla-Walker and Carlo, 2014). Thus, in addition to sharing, examining happiness after other types of prosocial behaviors would help to further investigate whether the emotional reward is a psychological mechanism that sustains several kinds of prosociality. Accordingly, the current study examined happiness after two additional types of prosocial behaviors (instrumental helping and empathic helping).

The link between empathic helping and happiness has not been investigated. As for instrumental helping, recently, using body posture as a measurement of positive emotion, researchers found that 2 years old show positive emotions after they helped an experimenter achieve her goal (Hepach et al., 2017a). However, in this study instrumental helping is a byproduct of play situations rather than an intentional behavior, as children had no chance to decide whether they wanted to help the experimenter; instead, they just happened to find the object that the experimenter needed during the session. Thus, it is still unclear whether and how these emotions change when children actually want to and succeed in helping others. Hence, more studies are needed to directly examine how emotions change when children initially intend and succeed in instrumental helping (Hepach et al., 2017a). In the current study we used facial expression to measure happiness, and we expected that if the emotional rewards of prosocial behaviors are ingrained in human nature, then most kinds of prosocial behavior should lead to an increase of happiness, not only sharing.

An alternative explanation for the increase of happiness is that it is the positive social feedback (such as being thanked or praised) after prosocial behaviors, but not the prosocial behaviors themselves, that promotes happiness. However, praising does not affect toddler’s instrumental helping subsequently (Warneken and Tomasello, 2008). Nevertheless, in order to further test this alternative explanation, more research on children’s happiness after prosocial behaviors and after receiving positive feedback is needed. Therefore, the current study evaluated toddler’s and prechooler’s happiness immediately after they performed instrumental helping or empathic helping behaviors, and after being thanked for this behavior. If happiness is a result of the social rewards/interactions after the prosocial behaviors, then happiness after being thanked would be higher than before they heard “thank you.” It should be noted that because we replicated Aknin’s methods for the sharing task (Aknin et al., 2012, 2015), in which the child was not thanked, we did not test this explanation in sharing.

## Current Study

The overarching goal of the current set of studies is to examine the link between prosocial behaviors and happiness for toddlers and young preschoolers. Specifically, we extend prior research by (1) replicating previous studies in two different cultures; (2) exploring whether and how resource availability would play a role in this (potential) relationship; (3) examining whether this positive relationship also exists for other prosocial behaviors (instrumental helping and empathic helping); and (4) examining the role of positive social feedback (i.e., being thanked) as an alternative explanation for the increase in happiness. For these purposes, we used a series of tasks to test Dutch toddlers’ (study 1), Dutch preschoolers’ (study 2) and Chinese preschoolers’ (study 3) prosocial behaviors and observe their happiness accordingly.

## Study 1

### Materials and Methods

#### Participants

Children were mainly recruited through daycares in several urban areas across the Netherlands, with nine participants recruited from posters in the university and word of mouth. After the daycare agreed to participate, the researchers sent active consent forms to the parents who had a toddler between 16 and 27 months of age. Parents were given a brief explanation of the tasks and told that the experiment would be conducted at the daycare center by two experimenters. In total, 122 toddlers were initially tested, with the majority being Dutch (95.9%), and coming from middle to upper middle-class families. Thirty-one toddlers did not show any of the targeted behaviors (sharing, instrumental helping, and empathic helping) in the experiment, leaving it impossible to measure their happiness while doing these behaviors. The study was approved by the Ethics committee of the Faculty of Social and Behavioral Sciences, Utrecht University. Informed written consent was obtained from all parents before the experiment. Previous studies had 20 children per sample (Aknin et al., 2012, 2015). In addition, *a priori* sample size calculation (using G^∗^Power 3.1.9.2, Faul et al., 2007) showed a total sample of 52 is needed to detect an effect size of 0.46 (with α at 0.05 level and 1 – β error prob at 0.90 level). Thus, the current sample size should be sufficient in detecting associations between happiness and prosocial behaviors.

#### General Procedure

The experiment was conducted in the child’s daycare (e.g., separate room or a separate corner of a larger room) by a main experimenter (E) and an assistant experimenter (AE). Both the E and AE arrived at the daycare at least 20 min before the testing and helped the teacher in arranging some classroom activities, so that the child could become familiar with them. Once the child felt comfortable with both the E and AE, he or she was invited to do the experiment. During the experiment, both the child and the E were videotaped. Neither the teachers nor parents were present during the testing, except for three cases where the children were too fussy to leave the teacher. In addition, the nine children that were recruited through posters and word of mouth were tested in either child’s daycare (*n* = 1), university lab (*n* = 1), or families’ home (*n* = 7). Parents were not present in the room during testing except for three cases where parents remained quiet and did not interact with the child. Each child completed a sharing, instrumental helping and empathic helping task, with the sharing task always coming first, following by the instrumental helping and empathic helping tasks in counterbalanced order. The session lasted approximately 10 min.

##### Sharing task (based on Aknin et al., 2012)

###### Warm up phase

The child got familiar with both the treats and the receiving/giving actions during the warm up phase. Each child met four stuffed animals (in order: mouse, rabbit, cat and panda) with their bowls in front of them, and were told that all animals “love to eat the treats.” Next, the E gave the child a bowl. Then the E gave each animal a treat, and the animal “ate” the treat by making eating sounds like “Yummm” and pushing the treat through a false bottom. After that, the E gave the child one treat, and asked the child whether he/she would like to eat the treat. In the next step, the E gave the child an extra bowl with five treats, and asked the child to share the treats with the four animals. The animal “ate” the treat when given. If, and only if the child hesitated in sharing, the E prompted him/her step by step (see Aknin et al., 2012 for detailed information). If the child shared with the animals, or if he/she refused to share after two rounds of prompts, the E put the animals (including their bowls) away, took away any remaining treats in the child’s bowl, and then moved to the formal test.

###### Test phase

The children were divided into three conditions (8, 4, or 2 treats they received in the test phase), balanced according to age and gender. The test included the following phases: (a) Meeting the monkey: The E introduced a new stuffed animal (Monkey) who also loves the treats, and encouraged but not forced the child to interact with the monkey (e.g., petting or touching). The E stressed that “now both you and the Monkey have no treats,” indicating to the child that the treats are a limited resource. (b) Receiving treats: The E “found” 2, 4, or 8 treats (depending on the condition), showed these treats to the child and put them in the child’s bowl while saying “Look, I found 2/4/8 treats, and I am giving them all to you”; (c) The E found one more treat and gave this treat to the Monkey; (d) The E found one more treat and asked the child to give this treat to the Monkey; (e) The E acted as if she could not find treats anymore, then asked the child to give one treat from his/her own bowl to the monkey. In order to prevent the order effects, phases (c)–(e) were performed in a counterbalanced order within each treat condition. For example, some toddlers were asked to share from their own resources first then to share from common pool. Non-costly or costly sharing behavior was identified if the child gave any treats from the common pool in phase d, or from his/her own bowl to the Monkey in phase e.

##### Instrumental helping and empathic helping (based on Svetlova et al., 2011)

In the instrumental helping task, the E wrapped five blocks one-by-one by using napkins that were placed on the table (one napkin within the reach of the child but not the E, and four within the reach of the E but not the child). After the E was out of the four napkins, she asked the child to hand her the final napkin by using eight sequential prompts (see Svetlova et al., 2011, for detailed information), and each prompt was present for 5–7 s. If the child helped, the E stopped providing prompts and took the napkin. After at least 3 s since the child helped, they said “thank you,” but did not give any other praise or rewards. Instrumental helping was identified if the child handed the napkin to the E before or directly after the last cue.

In the empathic helping task, the E showed the child a blanket, folded it around her shoulders, and stated “it makes me warm.” In this way, the link between the blanket and warmth was made explicit. Then the E put the blanket within the reach of the child while she was finding a toy bear, and let the child play with the bear. After 60 s, the E suddenly felt cold and shuddered, and prompted the child to hand the blanket to the E (similar prompts and time for each prompt as in the helping task). If the child helped the E by giving or pushing the blanket to her, the E stopped providing prompts and took the blanket. After at least 3 s since the child helped, they said “thank you,” but did not give any other praise or rewards. Empathic helping was identified if the child handed the blanket to the E before or directly after the last cue.

#### Coding Procedure

Two coders who were blind to the experimental hypotheses coded the children’s behavior (how many treats the child shared in phase e, and how many cues the child needed before instrumental/empathic helping). Intra-Class Correlations (ICC) were high, with a mean of 0.91 in coding behaviors (ICC _number shared_ = 0.98, ICC _steps before instrumental helping_ = 0.98, and ICC _steps before empathic helping_ = 0.77). The videos in which the children showed sharing, instrumental helping or empathic helping were given to new coders who only coded happiness using the same coding procedures as Aknin et al. (2012).

##### Happiness coding for children

We used the same rating scales that already had been used in various cultures (Canadian, Aknin et al., 2012; Vanatu, Aknin et al., 2015; and Chinese, Wu et al., 2017). In total five coders who were blind to the experimental hypotheses and the behavior coding rated the children’s happiness by using a 7-point scale *(1* = *not happy at all (very unhappy or crying), 2* = *big frown, 3* = *small frown, 4* = *neutral/flat affect, 5* = *small smile, 6* = *big smile, and 7* = *very happy/laughing)* (Aknin, personal communication, November 15, 2014).

For sharing, they coded the child’s happiness during phases (a)–(e). For instrumental helping and empathic helping, they coded happiness in the following three phases: (a) when the child watched the E wrapping the blocks (instrumental helping task)/showing the blanket (empathic helping task), prior to when the child was prompted to help; (b) when the child helped the E, but before the E said “thank you” (this phase lasted about 2 or 3 s); (3) after the E said “thank you” (this phase lasted about 2 or 3 s). The ICC for children’s happiness across coders was high, ranging from 0.85 to 1.00 for sharing, from 0.71 to 0.83 for instrumental helping and from 0.80 to 0.90 for empathic helping. Eighty-seven percent of the cases were coded by three coders, and the rest (13%) were coded by two coders. The happiness ratings represent the mean value across the coders.

##### Happiness coding for the E (and the Monkey they used)

Although all Es were blind to the experimental hypotheses and were trained to remain neutral during the experiment, another two raters coded the E’s/monkey’s “reaction” (combined happiness and enthusiasm) during the tasks (sharing, instrumental helping and empathic helping) in the same phases mentioned above for the children’s happiness rating, using the same 7-point scale (*1* = *not happy/enthusiastic at all, 4* = *neutral*, and *7* = *very happy/enthusiastic*). The two coders’ absolute agreement was high, ranging from 79.5 to 100% across all coding phases with a mean of 90.1%. The ICC could not be calculated, because the means of the coded values ranged from 3.99 to 4.17, and the *SD*’s from 0 to 0.46 on a 7-point scale. These results indicate that both the E and monkey showed a consistent, neutral emotion during the experiment.

### Results

#### Sharing

In total 73 of 122 participants shared one or more treats with the Monkey. *For those who shared*, three children’s happiness could not be coded in one or several of the phases as their faces moved out of the camera frame, and was excluded pairwise for the main analyses (including 2 cases in phase a, 1 in phase b, and 1 in phase d). In addition, *for those who did not share*, six children’s happiness could not be coded in phase a and b.

Preliminary analyses compared children who shared to those who did not on their levels of happiness when meeting the monkey (phase a) and receiving the treats (phase b). The independent samples *t*-tests showed that, compared with those who shared, children who did not share exhibited less happiness when meeting the monkey, *t*(111) = 2.11, *p* = 0.04, Cohen’s d effect size (*d*) = 0.41, and inclined to exhibit less happiness when receiving the treats, *t*(112) = 1.95, *p* = 0.05, *d* = 0.35.

**FIGURE 1:**
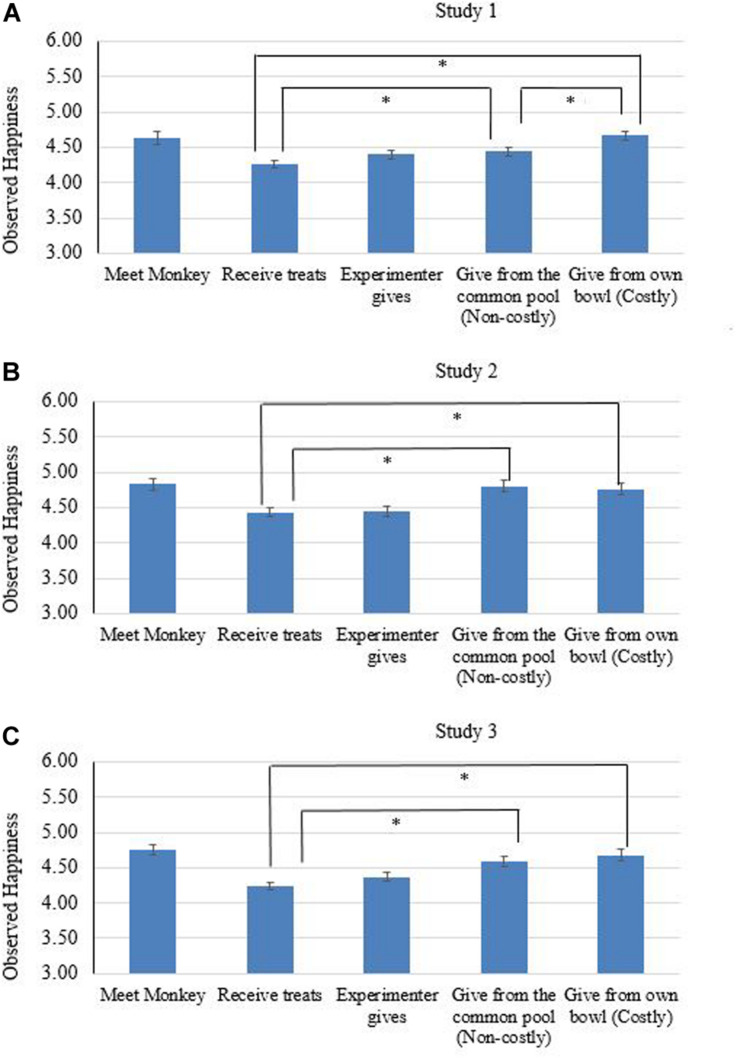
Observed happiness across the five phases in the sharing task, aggregated across all treat conditions. **(A)** presents the results for study 1, **(B)** for study 2, and **(C)** for study 3, respectively. Error bars display standard error of the mean. *^∗^p* < 0.05.

For the main research questions, we only included children who shared in the experiment. For the first research question (concerning whether toddlers show higher levels of happiness after sharing, compared with receiving treats), paired samples t-tests showed that, compared with receiving treats from E (phase b, *Mean*_b_ = 4.24, *SD* = 0.43, see Figure 1A), the participants expressed more happiness after they shared the treat from their own bowl (phase e, *Mean*_e_ = 4.67, *SD* = 0.53), *t*(70) = 3.08, *p* < 0.01, *d* = 0.83, and after they shared the treat from the common pool (phase d, *Mean*_d_ = 4.44, *SD* = 0.46), *t*(71) = 6.71, *p* < 0.01, *d* = 0.36. As for whether happiness is higher after costly than non-costly sharing, results showed children’s happiness to be higher in phase e than phase d, *t*(71) = 4.97, *p* < 0.01, *d* = 0.50.

For the second research question (whether the number of resources would affect the increase of happiness after costly sharing), we first calculated the difference between phase e and b for each child. Next, a one-way ANOVA test (across the 3 resource conditions) was conducted on the calculated difference. Results indicated no difference in children’s *increased* happiness (from phase b to e) among the different treat conditions, *F*(2, 71) = 1.99, *p* = 0.14^1^.

**FIGURE 2:**
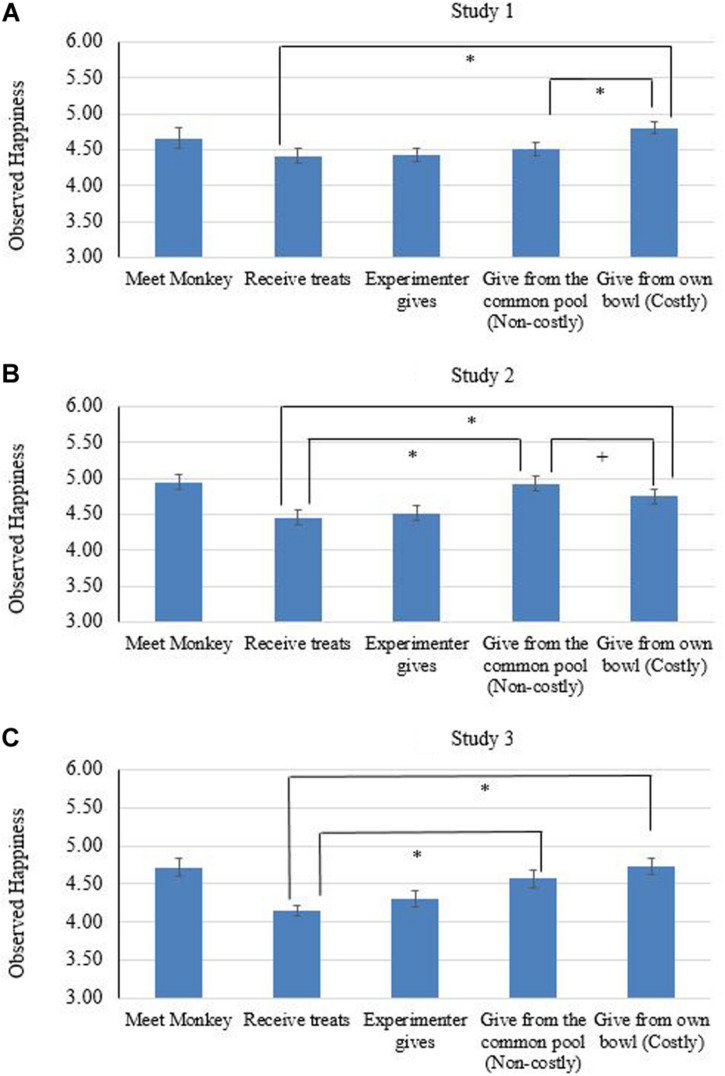
Observed happiness across the five phases in the sharing task, 8-treat condition. **(A)** presents the results for study 1, **(B)** for study 2, and **(C)** for study 3, respectively. Error bars display standard error of the mean. *^∗^p* < 0.05, ^+^*p* = 0.06.

In order to test whether we replicated the previous studies (Aknin et al., 2012, 2015), we analyzed data for the 8-treat condition only (*n* = 29). Paired samples *t*-tests showed that, compared with receiving treats from E (phase b, *Mean*_b_ = 4.41, *SD* = 0.53, see Figure 2A), the participants expressed more happiness after they shared the treat from their own bowl (phase e, *Mean*_e_ = 4.80, *SD* = 0.48), *t*(28) = 3.83, *p* < 0.01, *d* = 0.77, but not after they shared the treat from the common pool (phase d, *Mean*_d_ = 4.51, *SD* = 0.52), *t*(28) = 1.05, *p* = 0.30, *d* = 0.19). In addition, the participant expressed more happiness in phase e than in phase d, *t*(28) = 3.74, *p* < 0.01, *d* = 0.60, reflecting a higher level of happiness in a costly, compared with a non-costly sharing condition. Thus, we found the same pattern as previous studies. That is, sharing leads to an increase of happiness, and costly sharing leads to a greater increase in happiness compared with non-costly sharing.

Findings in the 4-treat condition were consistent with findings in the 8-treat condition. Compared with phase b, the participants expressed more happiness in phase e, *t*(20) = 4.68, *p* < 0.01, and in phase d, *t*(20) = 2.67, *p* = 0.02. Additionally, the happiness is higher in phase e than phase d, *t*(20) = 2.86, *p* = 0.01. Findings in the 2-treat condition revealed some different patterns. Similar to findings in the 8- and 4-treat condition, compared with phase b, participants expressed more happiness in phase e, *t*(21) = 3.33, *p* < 0.01. However, no difference was found between phase b and d, *t*(20) = 1.60, *p* = 0.13. Moreover, happiness was only (marginally) higher in phase e than phase d, *t*(20) = 1.98, *p* = 0.06.

#### Instrumental Helping and Empathic Helping

Because we counterbalanced the order between the instrumental helping and empathic helping tasks, we conducted an independent sample *t*-test to examine a possible task order effect. No significant differences were found, *p*_s_ > 0.24, indicating that there was no order effect on the happiness in these helping tasks. In total 87 participants engaged in instrumental helping and 66 participants engaged in empathic helping. *For those who helped*, one children’s happiness could not be coded in two phases (after they helped and being thanked) in the empathic helping task, as the face was blocked by the blanket. In addition, *for those who did not help*, 11 children’s happiness cannot be coded, including 11 cases in phase a in the instrumental helping task, and 5 cases in phase a in the empathic helping task.

##### Instrumental helping

**FIGURE 3:**
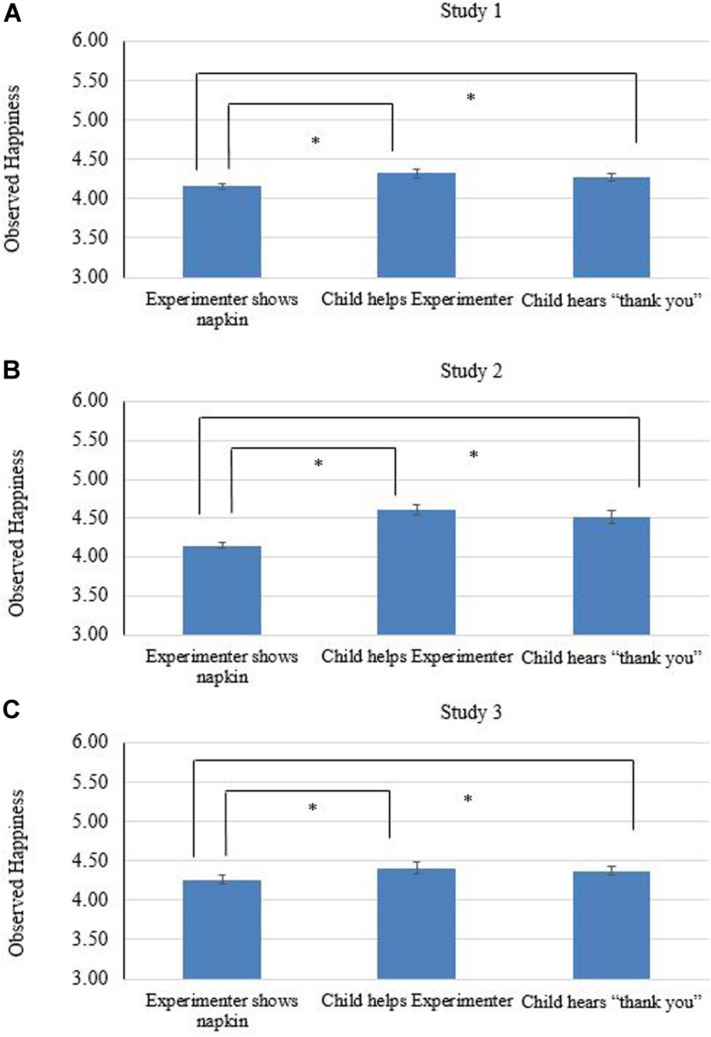
Observed happiness across three phases in the instrumental helping task. **(A)** presents the results for study 1, **(B)** for study 2, and **(C)** for study 3, respectively. Error bars display standard error of the mean. *^∗^p* < 0.05.

Preliminary analyses compared children who helped to those who did not help on their levels of happiness when watching the E wrapping the block (phase a), and found no differences, *t*(107) = 1.08, *p* = 0.28, *d* = 0.26. For the main research questions, we only included children who helped in the experiment. For the third research question (whether instrumental helping leads to an increase of happiness), paired samples t-tests showed that compared with watching E wrapping the block (phase a, *Mean*_*a*_ = 4.15, *SD* = 0.36, see Figure 3A), the participants expressed more happiness after they helped the E (phase b, *Mean*_*b*_ = 4.32, *SD* = 0.48), *t*(86) = 3.01, *p* = 0.00, *d* = 0.40, and after being thanked (phase c, *Mean*_*c*_ = 4.27, *SD* = 0.47), *t*(86) = 2.24, *p* = 0.03, *d* = 0.29. For the fourth research question (whether positive social feedback would lead to an increase of happiness after instrumental helping), paired samples t-test showed happiness did not differ between phase b and c, *t*(86) = 1.37 *p* = 0.17, *d* = 0.10.

##### Empathic helping

**FIGURE 4:**
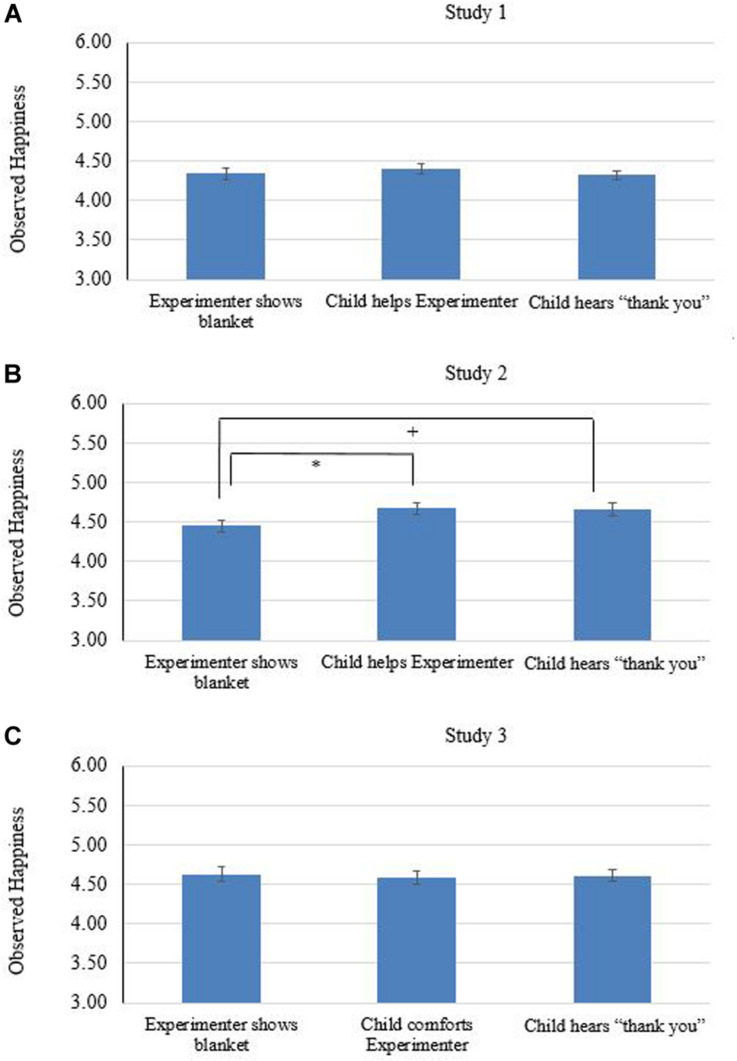
Observed happiness across the three phases in the comforting task k. **(A)** presents the results for study 1, **(B)** for study 2, and **(C)** for study 3, respectively. Error bars display standard error of the mean. *^∗^p* < 0.05, ^+^*p* = 0.06.

Preliminary analyses compared children who helped to those who did not help on their levels of happiness when watching E showing the blanket (phase a), and found no differences, *t*(112) = 1.41, *p* = 0.16, *d* = 0.28. For the main research questions, we only included children who helped in the experiment. For the third research question (whether empathic helping leads to an increase of happiness), paired samples t-tests showed that compared with witnessing E showing the blanket (phase a, *Mean*_*a*_ = 4.34, *SD* = 0.64, see Figure 4A), the participants showed the same level of happiness after they helped the E (phase b, *Mean*_*b*_ = 4.39, *SD* = 0.55), *t*(65) = 0.66, *p* = 0.52, *d* = 0.08), and after being thanked (phase c, *Mean*_*c*_ = 4.31, *SD* = 0.45), *t*(64) = 0.28, *p* = 0.77, *d* = 0.03). For the fourth research question (whether positive social feedback would lead to an increase of happiness after empathic helping), results showed happiness ratings did not differ between phase b and c, *t*(64) = 1.56, *p* = 0.12, *d* = 0.16.

#### Follow-Up Analyses on E’s Happiness Potentially Influencing Children’s Happiness

We further examined whether E’s happiness in each phase was related to children’s happiness in the same phase. For instance, in phase b for the sharing task, we analyzed the correlation between E’s happiness when giving the treats to the participants, and participants’ happiness when receiving the treats. If a significant correlation between E’s happiness and participants’ happiness was found, we re-ran the main analyses (using repeated measure analyses) involving that phase controlling for E’s happiness.

##### Results

For *sharing*, no significant correlations were found between E’s happiness and children’s happiness ratings in any phases, *p*_s_ > 0.23. Thus, E’ happiness does not appear to play a role in the findings on sharing. For *instrumental helping*, we found a correlation between E’s happiness and toddlers’ happiness when watching the E wrapping the block (phase a), *r* = 0.26, *p* = 0.02, but not after they helped the E (phase b), *p* = 0.11, or being thanked (phase c), *p* = 0.82. After controlling for E’s happiness in phase a, we still found that children exhibited more happiness in phase c than a, *p* = 0.02. However, the difference between phase a and b diminished to trend levels of significance, *p* = 0.08, indicating that E’s happiness appears to account for some but not all of the increase in children’s happiness after instrumental helping. For *empathic helping*, no significant correlations were found between E’s happiness and children’s happiness ratings in any phase. Thus, E’ happiness appears to not play a role in the findings on empathic helping. Overall, E’ happiness appears to not play a role in the main findings in study 1.

### Discussion

Thus, similar to previous studies, in study 1 we found sharing leads to a higher level of happiness after sharing, and higher levels of happiness in costly than non-costly sharing. Also, we replicated the main findings in previous studies for 8-treat condition. In addition, the increased of happiness after costly sharing did not differ based on number of treats received, implying the number of resources may not affect the link between sharing and happiness. Moreover, we found instrumental helping but not empathic helping to lead to higher levels of happiness. In order to examine whether this proposed mechanism is robust at different ages, in study 2 we further examined whether this warm-glow of prosocial behaviors remains when children are young preschoolers.

## Study 2

### Materials and Methods

#### Participants

One hundred and one of the participants (*M*_*age*_ = 33.99 months, *SD* = 3.96 months, 53 boys) in study 1 were tested again roughly 1 year later. There were no differences between the children who remained in the study in terms of their prosocial behaviors at Wave 1 nor on family demographics. Given that Study 1 and Study 2 involve using the same children, any differences in results are not likely to be a result of sample-selection differences.

#### General Procedure

The procedure was the same as in study 1.

#### Coding Procedure

The coding procedure largely followed that of study 1, except for two changes. First, two coders who were blind to the experimental hypotheses rated children’s happiness, including one coder (coder A) who rated all of the videos, and another coder (coder B) who rated 63% of the videos; the happiness ratings represent the mean value across the two coders for the 63% of the video, or the coding from coder A alone. The reliability between coders over the different phases was high (on average, ICC = 0.74). Second, only 1 coder coded E’s happiness.

### Results

#### Sharing

In total 79 of 101 participants shared one or more treats with the Monkey. *For those who shared*, 7 children’s happiness could not be coded in one or several of the phases in the sharing task, as their faces moved out of the camera frame (4 cases in phase a, 6 cases in phase b, 3 in phase d, and 3 in phase e). In addition, *for those who did not share*, 5 children’s happiness could not be coded (3 cases for phase a, 5 cases for case b).

Preliminary analyses compared children who shared to those who did not on their levels of happiness when meeting the monkey (phase a) and receiving the treats (phase b), and found no differences on happiness when meeting the monkey, *t*(95) = 1.23, *p* = 0.21, *d* = 0.32. Nevertheless, compared with those who shared, children who did not share showed less happiness when receiving the treats, *t*(91) = 1.95, *p* = 0.01, *d* = 0.65.

For the main research questions, we only included children who shared in the experiment. Compared with receiving treats from E (phase b, *Mean*_b_ = 4.45, *SD* = 0.60, see Figure 1B), the participants expressed more happiness after they shared the treat from their own bowl (phase e, *Mean*_e_ = 4.78, *SD* = 0.73), *t*(75) = 3.50, *p* < 0.01, *d* = 0.43, and after they shared the treat from the common pool (phase d, *Mean*_d_ = 4.81, *SD* = 0.78), *t*(86) = 4.73, *p* < 0.01, *d* = 0.52. However, there was no significant difference between phase e and phase d, *t*(77) = -1.67, *p* = 0.11, *d* = 0.12, indicating that across all conditions, children did not show a higher level of happiness after costly sharing compared with non-costly sharing. For the effect of the number of resources, a one-way ANOVA test found no differences in children’s *increased* happiness (from phase b to e) among the different treat conditions, *F*(2, 75) = 1.69, *p* = 0.19^2^.

For the 8-treat-condition, we found that, compared with receiving treats from E (phase b, *Mean*_b_ = 4.48, *SD* = 0.51), participants expressed more happiness after they shared a treat from their own bowl (phase e, *Mean*_e_ = 4.77, *SD* = 0.69, see Figure 2B), *t*(30) = 2.01, *p* = 0.04, *d* = 0.42, and after they shared a treat from the common pool (phase d, *Mean*_d_ = 4.96, *SD* = 0.79), *t*(32) = 3.82, *p* < 0.01, *d* = 0.72. In addition, the participant tended to express less happiness in phase e than in phase d, *t*(30) = -1.99, *p* = 0.06, *d* = 0.26, reflecting a lower level of happiness in a costly than a non-costly sharing condition.

Findings in the 4-treat condition were only partly consistent with findings in the 8-treat condition. Compared with phase b, participants again expressed more happiness in phase e, *t*(25) = 3.73, *p* < 0.01, and in phase d, *t*(25) = 2.46, *p* = 0.02. However, no difference was found between phase e than in phase d, *t*(26) = 0.70, *p* = 0.49. For the 2-treat condition, compared with phase b, participants expressed similar level of happiness in phase e, *t*(18) = 1.60, *p* = 0.13, and in phase d, *t*(18) = 0.43, *p* = 0.67. In addition, the participant expressed less happiness in phase e than in phase d, *t*(19) = -2.33, *p* = 0.03, reflecting a lower level of happiness in a costly than a non-costly sharing condition.

#### Instrumental Helping and Empathic Helping

Again we conducted an independent sample t-test to examine a possible task order effect, and no significant differences were found, *p*_s_ > 0.12, indicating that there was no order effect on the happiness in these helping tasks. In total 84 participants engaged in instrumental helping and 80 participants engaged in empathic helping. However, *for those who helped*, 3 children’s happiness could not be coded in phase c in instrumental helping, and 4 children’s happiness could not be coded in one or several of the phases in empathic helping task (1 case in phase a, and 3 in phase c), as their faces moved out of the camera frame or were blocked by the blanket. In addition, for those who did not help, 6 children’s happiness could not be coded, including 5 cases in phase a in the instrumental helping task, and 5 cases in phase a in the empathic helping task.

##### Instrumental helping

Preliminary analyses compared children who helped to those who did not help on their levels of happiness when watching E wrapping the block (phase a). Compared with those who helped, children who did not help showed less happiness, *t*(96) = 2.21, *p* = 0.04, *d* = 0.66. For the main research questions, compared with watching E wrapping the block (phase a, *Mean*_*a*_ = 4.18, *SD* = 0.35, see Figure 3B), the participants expressed more happiness after they helped the E (phase b, *Mean*_*b*_ = 4.61, *SD* = 0.64), *t*(83) = 6.64, *p* < 0.01, *d* = 0.83, and after being thanked (phase c, *Mean*_*c*_ = 4.53, *SD* = 0.67), *t*(80) = 5.14, *p* < 0.01, *d* = 0.64. However, happiness did not differ between phase b and c, *t*(80) = 1.58, *p* = 0.12, *d* = 0.13.

##### Empathic helping

Preliminary analyses compared children who helped to those who did not help on their levels of happiness when watching E showing the blanket (phase a), and found no differences, *t*(93) = 1.10, *p* = 0.27, *d* = 0.30. For the main research questions, we only included children who helped in the experiment. Compared to witnessing E showing the blanket (phase a, *Mean*_*a*_ = 4.45, *SD* = 0.75, see Figure 4B), the participants tended to show higher level of happiness after they helped the E (phase b, *Mean*_*b*_ = 4.66, *SD* = 0.71), *t*(78) = 2.21, *p* = 0.05, *d* = 0.28, and more happiness after being thanked (phase c, *Mean*_*c*_ = 4.64, *SD* = 0.70), *t*(75) = 1.89, *p* = 0.06, *d* = 0.23. In addition, happiness ratings did not differ between phase b and c, *t*(76) = 0.38, *p* = 0.71, *d* = 0.03.

#### Follow-Up Analyses on E’s Happiness Potentially Influencing Children’s Happiness

For *sharing*, no significant correlations were found when receiving treats from the E (phase b), or sharing treats from their own bowl (phase e). Thus, E’s happiness appears to not play a role in the findings that preschoolers exhibited more happiness in phase e than b. In addition, we found a correlation between E’s and children’s happiness when sharing treats from the common pool (phase d), *r* = 0.25, *p* = 0.03. After controlling for E’s happiness in phase d, results revealed two differences to the main findings. First, the difference on the happiness between phase b and d reduced from significant to non-significant, *p* = 0.16. Thus, E’s happiness might explain the increased happiness after sharing treats from the common pool than receiving treats from the E. Second, we found a new trend; that is, participants expressed more happiness in phase e than phase d, *p* = 0.07, showing that they expressed more happiness after sharing from their own resources than from the common pool.

For *instrumental helping*, no correlations were found between E’s happiness and children’s happiness in any phases, *p*_s_ > 0.16. Thus, E’ happiness appears to not play a role in the findings on instrumental helping.

For *empathic helping*, no correlations were found between E’s happiness and children’s happiness in witnessing the E showing the blanket (phase a), *p* = 0.17, or they helped the E (phase b), *p* = 0.21. Thus, E’ happiness appears to not play a role in the findings that preschoolers exhibited more happiness in phase b than a. In addition, only a marginally significant correlation was found after being thanked (phase c), *p* = 0.07. After controlling for E’s happiness in phase c, we still found no difference on toddlers’ happiness between phase a and c, nor between phase b and c, *p*_s_ > 0.29. Thus, E’ happiness appears to not play a role in the findings on empathic helping.

Overall, E’s happiness appears to not play a role in the main conclusions drawn in study 2, except that preschoolers may tend to exhibit more happiness after costly than non-costly sharing, but no longer exhibited more happiness after non-costly sharing than receiving treats.

### Discussion

Similar to study 1 and previous studies, we found that preschoolers exhibited more happiness after sharing than receiving treats. However, different from previous studies, we did not find a difference between costly and non-costly sharing, even in the 8-treat condition, and there was a trend that preschoolers were less happy in costly than non-costly sharing. For instrumental helping, we found similar results in terms of happiness as in study 1, showing that helping leads to a higher level of happiness. For empathic helping, different from study 1, children showed higher levels of happiness after helping the experimenter. Thus, the warm-glow of prosocial behavior continues to be found as children move into the preschool ages. In order to further test the universality of the links between prosocial behaviors and happiness, in study 3 we examined these relationships among Chinese preschoolers.

## Study 3

### Materials and Methods

#### Participants

Participants were recruited from two daycares in Shanghai, China. The recruitment procedure was the same as in study 1. In total 91 Chinese preschoolers (*M* = 48.54 months, *SD* = 6.15 months, 43 boys) were tested, with the majority coming from middle to upper middle-class families. All children at least shared or helped once in the experiment.

#### General Procedure

The procedure was same as in study 1 and 2.

#### Coding Procedure

The procedure was same as in study 2, except that two coders (same coders as for study 2) rated all children’s happiness, and the happiness ratings represent the mean value across the two coders. The reliability between coders over the different phases was high (on average, ICC = 0.85). Additionally, followed that of study 1, two coders coded E’s happiness (66.1 and 46.1% of the videos). The absolute agreement was high, with a mean of 95.26%.

### Results

#### Sharing

In total 88 preschoolers shared in the experiment. However, *for those who shared*, 5 children’s happiness could not be coded in one or several of the phases in the sharing task, as their faces moved out of the camera frame (1 case in phase b, and 4 cases in phase d).

Preliminary analyses compared children who shared to those who did not on their levels of happiness when meeting the monkey (phase a) and receiving the treat (phase b), and found no differences on happiness when meeting the monkey, *t*(89) = 1.11, *p* = 0.27, *d* = 0.65. Nevertheless, compared with those who shared, children who did not share showed less happiness when receiving the treats, *t*(86) = 0.88, *p* < 0.01, *d* = 0.51.

For the main research questions, we only included children who shared in the experiment. Compared with receiving treats from E (phase b, *Mean*_b_ = 4.24, *SD* = 0.47, see Figure 1C), the participants expressed more happiness after they shared the treat from their own bowl (phase e, *Mean*_e_ = 4.68, *SD* = 0.71), *t*(86) = 6.38, *p* < 0.01, *d* = 0.70, and after they shared the treat from the common pool (*Mean*_d_ = 4.60, *SD* = 0.65), *t*(83) = 6.72, *p* < 0.01, *d* = 0.63. However, they tended to show more happiness in phase e than d, *t*(83) = 1.87, *p* = 0.07, *d* = 0.12.

For the effect of the number of resources, a one-way ANOVA test showed no difference in children’s *increased* happiness (from phase b to e) among the different treat conditions, *F*(2, 86) = 1.58, *p* = 0.21^3^.

For the 8-treat condition, compared with receiving treats from E (phase b, *Mean*_b_ = 4.15, *SD* = 0.35, see Figure 2C), children expressed more happiness after they shared the treat from their own bowl (phase e, *Mean*_e_ = 4.72, *SD* = 0.58), *t*(28) = 5.98, *p* < 0.01, *d* = 1.19, and after they shared the treat from the common pool (phase d, *Mean*_d_ = 4.57, *SD* = 0.63), *t*(27) = 4.60, *p* < 0.01, *d* = 0.80. However, the participants expressed similar level of happiness in phase e and phase d, *t*(27) = 1.43, *p* = 0.17, *d* = 0.21.

Findings in the 4- and 2-treat conditions were consistent with findings in the 8-treat condition. Compared with phase b, the participants tended to express more happiness in phase e [4-treat condition, *t*(29) = 2.07, *p* = 0.05; 2-treat condition, *t*(27) = 4.08, *p* < 0.01], and after they shared the treat from the common pool [4-treat condition, *t*(27) = 2.47, *p* = 0.02; 2-treat condition, *t*(26) = 2.35, *p* < 0.01]. However, the participants expressed similar levels of happiness in phase e and phase d [4-treat condition, *t*(27) = 1.35, *p* = 0.19; 2-treat condition, *t*(27) = 0.40, *p* = 0.69].

#### Instrumental Helping and Empathic Helping

Again we conducted an independent sample *t*-test to examine a possible task order effect, and no significant differences were found, *p*_s_ > 0.34, indicating that there was no order effect on the happiness in these helping tasks. In total 84 participants engaged in instrumental helping and 83 participants engaged in empathic helping. However, *for those who helped*, 1 child’s happiness could not be coded in phase b in instrumental helping, and 1 child’s happiness could not be coded in phase b in the empathic helping task, as their faces moved out of the camera frame.

##### Instrumental helping

Preliminary analyses compared children who helped to those who did not help on their levels of happiness when watching E wrapping the block (phase a). Compared with those who helped, children who did not help tended to show less happiness, *t*(9) = 1.98, *p* = 0.08, *d* = 0.42. For the main research questions, compared with watching E wrapping the blocks (phase a, *Mean*_*a*_ = 4.27, *SD* = 0.47, see Figure 3C), the participants tended to express more happiness after they helped the E (phase b, *Mean*_*b*_ = 4.41, *SD* = 0.64), *t*(82) = 2.00, *p* = 0.05, *d* = 0.23, but expressed similar levels of happiness after being thanked (phase c, *Mean*_*c*_ = 4.38, *SD* = 0.57), *t*(83) = 1.68, *p* = 0.97, *d* = 0.21. In addition, happiness did not differ before and after they heard “thank you,” *t*(82) = 0.88, *p* = 0.38, *d* = 0.05.

##### Empathic helping

Preliminary analyses compared children who helped to those who did not help on their levels of happiness when watching the E showing the blanket (phase a), and found no differences, *t*(89) = 0.49, *p* = 0.53, *d* = 0.16. For the main research questions, compared to witnessing E showing the blanket (phase a, *Mean*_*a*_ = 4.63, *SD* = 0.84, see Figure 4C), the participants showed the same level of happiness after they helped the E (phase b, *Mean*_*b*_ = 4.59, *SD* = 0.76), *t*(81) = 0.58, *p* = 0.57, *d* = 0.07, and after being thanked (phase c, *Mean*_*c*_ = 4.59, *SD* = 0.71), *t*(82) = 0.48, *p* = 0.63, *d* = 0.05. In addition, happiness ratings did not differ before and after they heard “thank you,” *t*(81) = -1.73, *p* = 0.86, *d* = -0.04.

#### Follow-Up Analyses on E’s Happiness Potentially Influencing Children’s Happiness

For *sharing*, we found (marginally) significant correlations between E’s happiness and children’s happiness when receiving treats (phase b), *r* = 0.25, *p* = 0.02; after they shared the treat from the common pool (phase d), *r* = 0.22, *p* = 0.05; and after they shared the treat from their own bowl (phase e), *r* = 0.28, *p* = 0.01. After controlling for E’s happiness, results revealed three differences to the main findings, reducing the effects from significant to non-significant. First, no difference on preschoolers’ happiness was found when comparing phase b and phase e, *p* = 0.95; second, no difference was found between phase b and phase d, *p* = 0.77; and finally, no difference was found phase d and phase e, *p* = 0.71. Thus, E’s happiness accounted for the increase in children’s happiness after they shared the treat from their own bowl compared to when receiving treats, after they shared from the common pool than when receiving treats, and after they shared from their own bowl compared to when they shared from the common pool.

For *instrumental helping*, we found significant correlations between E’s happiness and children’s happiness when watching the E wrapping the block (phase a), *r* = 0.23, *p* = 0.04, and helping E (phase b), *r* = 0.31, *p* = 0.01. However, no correlation was found after being thanked (phase c), *r* = 0.11, *p* = 0.35. After controlling for E’s happiness, we still found that, compared with phase a, the participants expressed more happiness in phase b, *p* = 0.02, and in phase c, *p* = 0.03. In addition, we found a new trend, that is, the participants exhibited more happiness in phase b than c, *p* = 0.04.

For *empathic helping*, we found significant correlations between E’s happiness and preschoolers’ happiness when witnessing the E showing the blanket (phase a), *r* = 0.36, *p* < 0.01, and after they helped (phase b), *r* = 0.24, *p* = 0.04. However, no correlation was found after being thanked (phase c), *r* = 0.20, *p* = 0.09. After controlling for E’s happiness, we still found that, compared with phase a, the participants exhibited similar levels of happiness in phase c, *p* = 0.81, and happiness ratings did not differ in phase b and c, *p* = 0.40. In addition, we found a new trend, that is, compared with phase a, the participants exhibited more happiness after they helped (phase b), *p* = 0.05. Thus, compared with witnessing the E showing the blanket, preschoolers tended to exhibit more happiness after they helped.

In order to examine the cross-cultural validity of the current findings on E’s happiness, we compared the happiness exhibited between the Chinese and Dutch experimenters. In the sharing task, the Chinese experimenters showed a higher level of happiness than the Dutch experimenters in all phases (*p*_s_ < 0.01, Phase b, *Mean difference*_(Chinese – Dutch)_ = 0.16; Phase d, *Mean difference*_(Chinese – Dutch)_ = 0.20; Phase e, *Mean difference*_(Chinese – Dutch)_ = 0.19). In the instrumental helping task, they showed similar levels of happiness in phase a (*p* = 0.50) and b (*p* = 0.17), but higher levels of happiness than the Dutch experimenters in phase c (*p* < 0.01, *Mean difference*_(Chinese – Dutch)_ = 0.68). In the empathic helping task, they showed higher level of happiness in all phases (*p*_s_ < 0.05, Phase a, *Mean difference*_(Chinese – Dutch)_ = 0.32; Phase b, *Mean difference*_(Chinese – Dutch)_ = 0.12; Phase c, *Mean difference*_(Chinese – Dutch)_ = 0.62).

### Discussion

Overall, in the Chinese sample we found that preschoolers’ happiness was related to E’s happiness in most of the phases tested. In addition, although Chinese preschoolers also showed higher levels of happiness after sharing than receiving treats in the main analyses, these effects were no longer significant after controlling for E’s happiness. Nevertheless, similar to our findings in Dutch preschoolers, we found higher levels of happiness after instrumental helping, both before and after controlling for E’s happiness. In addition, after controlling for E’s happiness, higher levels of happiness were found after empathic helping, similar to the findings in Dutch preschoolers.

## General Discussion

The current study aimed to (1) replicate previous studies about the emotional rewards of sharing in toddlers; (2) examine whether the emotional rewards are affected by the number of resources available before sharing; (3) explore the possible emotional rewards of instrumental helping and empathic helping and (4) investigate the possible role of positive social feedback by comparing toddlers’ happiness before and after being socially rewarded. Overall, these four aims help to explore whether toddlers and preschoolers experience prosocial behavior to be emotionally rewarding. In general, results revealed that Dutch toddlers (study 1), Dutch preschoolers (study 2), and Chinese preschoolers (study 3) are happier when having just shared or helped and this happiness does not depend on the number of resources the child had or whether the child was thanked for the behavior. When the experimenters’ happiness was taken into account as a possible confound, the effect for sharing for Chinese preschoolers disappeared.

Regarding the first aim, when comparing the happiness between sharing and receiving treats, we found that young children in the Netherlands and China were happier after sharing from the common pool (non-costly sharing) compared with receiving treats. However, compared with previous studies that found large effect sizes (Aknin et al., 2012, 2015), only small to medium effect sizes were found in the current study. Moreover, we found young children in both cultures were happier after sharing their own treats (costly sharing) with a monkey compared to receiving treats. In line with previous studies (Aknin et al., 2012, 2015), we found medium to large effect sizes on the increased happiness after costly sharing. These results further support the idea that the warm glow is a universal mechanism that sustains sharing behaviors at early ages consistently, and especially when these young children really need to sacrifice their own resources (i.e., costly sharing).

In addition to comparing happiness between sharing and receiving, we also compared the happiness between two types of sharing behaviors, in order to examine whether costly sharing is more emotionally rewarding than non-costly sharing. Only toddlers, but not preschoolers, showed that costly sharing is more rewarding than non-costly sharing. Specifically, consistent with previous studies (Aknin et al., 2012, 2015), Dutch toddlers were happier during costly sharing (sharing from their own resources) than non-costly sharing (sharing from the common pool). Also, Chinese preschoolers were inclined to show more happiness in costly than non-costly sharing. In contrast, Dutch preschoolers were inclined to show less happiness in costly than non-costly sharing. These findings imply that even though sharing, both non-costly and (especially) costly sharing, is initially emotional rewarding, other factors may gradually interact with this mechanism during the preschool age. A potential candidate is the development of ownership understanding. Indirect evidence lies in the studies on the relationship between ownership understanding and sharing behavior between the ages of two to four. Specifically, ownership understanding is positively related to costly sharing in toddlerhood (e.g., Hay, 2006; Brownell et al., 2013), but is negatively related during the early preschool ages (Eisenberg-Berg et al., 1979, 1981; Friedman and Neary, 2008). According to the current findings, young preschoolers may gradually find costly sharing to be equal to or even less rewarding than non-costly sharing, and may thus be less inclined to share what they already had. In this way, with the development of ownership understanding, individuals may gradually become more “strategic,” namely, to both protect their own goods and to fulfill others’ material needs. Nevertheless, more studies are needed to directly examine how ownership understanding may or may not contribute to the emotional benefits of prosocial behaviors during development.

Regarding the second aim, the current results did not show a resource effect on the increase of happiness after costly sharing compared with receiving treats in either culture and/or age group examined. These findings further support the idea that the warm glow of giving is independent of resource availability at these ages. Nevertheless, in the current study, children were asked to share their windfalls (i.e., treats), and we did not directly measure their preference for the resources. Thus, their desires for these treats are unclear. In order to further tease apart the effect of children’s own desire for the resources in relation to the emotional rewards of sharing, further studies can manipulate both the quantity (e.g., the number of resources) and the quality (e.g., the preference for resources) of the materials used in the experiment. For example, they could ask children to share something they already own (i.e., their own toys).

Regarding the third aim, it was found that happiness also increased after instrumental helping behavior, and the effect sizes were medium to large (*d* ranged from 0.40 to 0.82). These findings support the idea that the internal emotional reward of acting prosocially is not a sharing-specific mechanism, but exists in different kinds of prosocial behaviors. For empathic helping, findings were less consistent. While no increase in happiness was found after empathic helping for Dutch toddlers, Dutch preschoolers showed an increase of happiness after empathic helping. Also, only after controlling for E’s happiness, Chinese preschoolers were inclined to show an increase of happiness after empathic helping. Several explanations are possible for the non-significant increase of happiness after empathic helping. First, the children might have felt “interrupted” in their play with the bear, thereby having to switch their attention from the toy to the experimenter, while in the other tasks the child was already focused on the activity of the experimenter. Second, the absence of higher levels of happiness might also be related to the distress expressed by the experimenter. Already 2 years old can show sympathetic arousal after observing a stranger in distress (Hepach et al., 2012). It may take longer for the child’s positive emotions to kick in after observing the experimenter’s distress. In addition, different explanations may relate to studies 1 and 3, respectively. For Dutch toddlers (study 1), this task might be cognitively challenging with too many inferential steps (Warneken and Tomasello, 2014), that perhaps overrides any emotional response to the task. During the task, they need to notice the experimenter’s feeling (cold) and her need (to keep warm), recall the relationship between the blanket and warmth, and hand over the right object (the blanket, not the bear). As for Chinese preschoolers (study 3), they might be regulating their own emotions in order to not show happiness in front of a distressed person (Vaish et al., 2009). After all, after controlling for experimenters’ happiness, these children indeed showed more happiness after empathic helping. However, more research on age and cultural differences in emotion regulation and happiness after empathic helping behaviors are needed before any conclusions can be drawn.

Regarding the fourth aim, we found that toddlers’ happiness remained the same after being thanked in both the instrumental helping and empathic helping tasks in both cultures, suggesting that happiness is the likely result from prosocial behaviors, and not the positive social feedback from the experimenter (i.e., being thanked). Otherwise, children would have shown a higher level of happiness after being thanked. Thus, the findings that an increase of happiness occurs after sharing and instrumental helping, aligns with previous research that speaks to the intrinsic motivation of prosocial behaviors. For example, toddlers showed greater internal arousal after witnessing strangers’ needs being fulfilled even though they were unable to help the stranger (Hepach et al., 2016), indicating they have a genuine concern for others’ needs. In addition, toddlers helped when there is no possibility for future rewards (Hepach et al., 2017b), and their helping behaviors are not affected, or even undermined by social enforcement (Warneken and Tomasello, 2008).

In addition to these findings, several questions remain. First, after taking the experimenter’s happiness into account, for Dutch preschoolers, we found the warm glow of three types of prosocial behaviors (i.e., sharing, instrumental helping, and empathic helping). In comparison, for Chinese preschoolers, we found this warm glow of empathic helping and instrumental helping, but not for sharing. The experimenter’s happiness seems to fully explain the happiness increase after sharing for Chinese preschoolers. There are at least three possible explanations for this effect. First, compared with the Dutch experimenters, the Chinese experimenters showed a slightly higher level of happiness than the Dutch experimenters in all phases of the sharing task. Accordingly, the different patterns found in two cultures may actually result from between-experimenter differences in exhibited happiness. Future studies should strive for more control over the experimenter’s emotional expression, ideally by employing bi-lingual experimenters to conduct the tasks in both cultures, though at least by providing more stringent training on maintaining a neutral state. A second, alternative, explanation might be that, at this age, social/cultural factors beyond sharing behavior itself may also contribute to the explanation of the warm glow effect. For instance, given that parents from individualistic cultures emphasize relational socialization goals set for their children less often than those from collectivistic cultures (e.g., Giner Torréns and Kärtner, 2017), and that the Dutch culture is more individualistic than Chinese culture^4^, the Chinese preschoolers we tested may be more sensitive, and be more inclined to respond to and/or mimic E’s emotion. Indeed, in the sharing task, Chinese preschoolers’ happiness related to E’s happiness in three phases examined in the current study. In comparison, Dutch preschoolers’ happiness related to E’s happiness in only one phase. Thus, the happiness increase after sharing may (partly) be explained by a reaction toward the recipient in the Chinese culture. Finally, it could also be that Chinese experimenters responded/mimicked preschoolers’ happiness. If this was the case, then the Chinese preschoolers may have experienced the same warm glow of sharing as the Dutch preschoolers. However, since experimenters asked participants to share before they actually shared, this is a less likely explanation based on the current experimental design. Nevertheless, because the current findings are correlational, more studies are needed to further distinguish the second and the third explanations.

Moreover, despite finding several significant effects, practical implications of these findings are unclear. For instance, all the happiness ratings fell between “neutral state” (= 4) and “small smile” (= 5) in the current study. Thus, even though the warm glow existed, both the recipients and the children who shared/helped may not perceive this increase of happiness. That is, the warm glow may sustain prosocial behavior implicitly/unconsciously. Nevertheless, we only used observed assessments of children’s happiness as the only measure of children’s positive affect. Different indices have been developed in measuring child’s positive emotions (e.g., using upper-body posture; Hepach et al., 2017a). Further studies could use different measurements of positive emotions after performing prosocial behaviors, which could help to better understand how people perceive the “warm glow” of prosocial behaviors.

It is also noteworthy that, extra caution should be addressed to the generalizability and validity of the current findings. First, participants’ age and number of resources available for sharing should be taken into account when interpreting the results. This is especially the case for studies 1 and 2, where we found only 60% of the toddlers shared (study 1), and 78% of the preschoolers shared (study 2). In previous studies, almost all participants shared (Aknin et al., 2012, 2015). The resource conditions we added may have lad to a lower percentage of sharing. That is, while previous studies used an 8-treat condition, we used 2-, 4-, and 8- treat conditions. Indeed, participants were less likely to share in the 2- and 4-treat conditions, compared with the 8-treat condition (Song, 2019). In addition, the age tested may also explain the lower percentage of sharing in study 1. The original task was designed and conducted for toddlers who are at least 22 months, while in the current study, 43.8% of the participants were younger than 22 months. We found that children younger than 22 months were indeed less likely to share than their older peers in the current task (Song, 2019), implying that the current task may be too complex for these young children.

Second, the current findings on the warm glow of prosocial behaviors are only for those who actually engaged in prosocial behaviors. More studies are needed to further test *individual differences* in experiences of the warm glow. More specifically, for those who did not engage in prosocial behaviors, we found that they were less happy in the beginning of the task (e.g., when meeting the monkey/receive treat in the sharing task, or meeting the experimenter in the helping tasks), compared with children who actually shared/helped. As mentioned above, in Aknin’s studies, almost all participants shared (Aknin et al., 2012, 2015), leaving it not possible to compare the happiness for those who shared and those who did not. In addition, no difference on happiness before sharing was found by Wu et al. (2017). In the current study, the lower level of happiness may indicate that these children were either not interested in the task at the beginning or more timid or shy than other children. It may also related to the temperament of children. Shyness, for instance, is negatively related to prosocial behaviors. Another explanation, related to the “positive feedback loop” (Aknin et al., 2018, p. 55), states that prosocial behaviors lead to an increase of happiness, which further sustains children to engage in prosocial behavior again. Thus, the more children engaged in prosocial behaviors, the more emotional rewards, and (maybe) the more enthusiastic/happy they are when interacting with others in the first place. Overall, it is plausible that, depending on their initial level of happiness, some individuals may be more affected by this warm glow of happiness than others. The current study cannot speak to this possibility. We mainly focused on the toddlers/preschoolers who helped, and/or shared in the tasks, comparing whether happiness changes before and after doing so. We did not compare whether happiness changes among children who did not help or share and therefore are unable to say whether happiness is affected by not helping or sharing. Thus, future studies are needed to examine whether happiness (or other emotions) change among those who do not help/share.

The current study has some limitations that should be considered in future research. First, methodologically, although all the coders were blind to our research questions and experimental expectations, they are not blind to the phases (e.g., phase a to e in the sharing task) when coding children’s happiness. Thus, their coding might still be biased by the happiness shown at other points during the study (Fast, 2018), and/or by the on-going interactions between the child and the experimenter. A more appropriate way for coding children’s happiness is to provide short, audio-free clips, in which only the child’s face in each phase was displayed for coders, and the order for presenting these clips should be pre-determined by a new research assistant (Aknin et al., 2015; Fast, 2018). Nevertheless, in the current study, we used two cameras to videotape the child and the experimenter separately and we also controlled for E’s happiness in analyses. In this way, at least we can partly tease apart the influence of the experimenter’s on children’s coded happiness. A second limitation is the low power of some analyses. This is especially the case when examining the emotional rewards of sharing for each treat-condition. More specifically, in either the 2-, 4-, or 8-treat condition, the sample sizes were less than 30. Thus, extra caution should be taken when interpreting the null findings of these analyses. Third, for the design of the current study, the order of the tasks is partly fixed. Because we wanted to see whether we could replicate previous findings in sharing, we always presented sharing tasks first, and then the two helping tasks in the counterbalanced order. It is plausible that children are experienced diminishing marginal utility of additional prosocial opportunities. For instance, they may be accustomed to, or even bored with engaging in helping. Accordingly, they might be less likely to help, and more importantly, even if they helped, the increase of happiness after helping may diminish. However, we did not find the order effects of two helping tasks on children’s happiness in helping tasks. These findings suggest that children’s happiness in the helping tasks should (at least) not be affected by the order between the two helping tasks. Nevertheless, in order to further tease apart the order effect, further studies should counterbalance all three tasks. Fourth, in the current study we asked the children to behave prosocially. Thus, happiness might result from compliance to adults. Although it is impossible to rule out this explanation based on the current experimental design, the differences in happiness between costly and non-costly sharing suggest that compliance in and of itself cannot fully explain these results. After all, in both conditions, they are complying to experimenters’ requests. Moreover, as shown by Wu et al. (2017), obligated sharing does not results in increases in happiness. Nevertheless, further studies should directly measure children’s happiness after spontaneous prosocial behaviors, in order to further tease apart the confounding effects of compliance. Fifth, prosocial behavior examined in the current study always involved active contact with the experimenter. It is plausible that, children’s social interactions, regardless of being prosocial or not, contribute to the increased happiness. Accordingly, further studies should examine this alternative explanation by manipulating the outcome of children’s actions (prosocial or non-prosocial) such as asking children to either share objects or simply re-locate objects. Sixth, in the current study we used “thank you” as an index of social rewards. It is commonly used in daily activities when praising children after prosocial behaviors, however, whether this kind of praise is strong enough for making a difference in the level of happiness needs further exploration. Finally, the findings in the sharing task may be confounded with order effects. Specifically, the phase of receiving treats always comes before children are asked to share. An improvement to the current design would be to counterbalance the order of receiving and sharing by adding a phase in which the child is asked to share something he/she already owned. Specifically, children participate in four trials in counterbalanced order (in each trial, they meet a new puppet, so that the activity in one trial would been seen as independent of the other trials), including (a) only receiving some windfalls, (b) sharing from the common pool, (c) sharing something from what they owned before the experiment (e.g., items they received the day before), and (d) receiving and sharing some windfalls.

## Conclusion

In conclusion, the current study adds more evidence supporting the universality of emotional rewards of prosocial behaviors by demonstrating that both Dutch and Chinese young children exhibited more happiness after sharing with and instrumentally helping a stranger. Furthermore, we did not find evidence to support the effect of resource availability or positive social feedback (i.e., being thanked) on happiness. However, it seems that not all kinds of prosocial behaviors are emotionally rewarding at all ages, as we found inconsistent results for empathic helping. Thus, future work can further examine at what age, and how this warm glow occurs under different conditions and various forms of prosocial behaviors.

## Data Availability Statement

The datasets generated for this study are available on request to the corresponding author.

## Ethics Statement

The studies involving human participants were reviewed and approved by the ethical committee of social and behavior science school, Utrecht University. Written informed consent to participate in this study was provided by the participants’ legal guardian/next of kin.

## Author Contributions

YS conceptualized the study, trained undergraduate and graduate students who collected and coded the data, analyzed the data, and wrote the manuscript. MB and JD gave advice and feedback on study design, data collection, data analyses, and manuscript. All authors contributed to the article and approved the submitted version.

## Conflict of Interest

The authors declare that the research was conducted in the absence of any commercial or financial relationships that could be construed as a potential conflict of interest.

## Footnotes

1 Given the small sample for each resource condition, we also conducted Bayesian analyses to examine the potential treat effect on the increase in happiness, and the conclusions remained the same. More specifically, we tested two models: Model A (*M*_2-treat_ = *M*_4-treat_ = *M*_8-treat_); and Model B (*M*_2-treat_ < *M*_4-treat_ < *M*_8-treat_). We used the R package (R Core Team, 2013) “Bayesian informative hypothesis testing” (*Bain*: Mulder, 2014; Gu et al., 2019) to assess the relative evidence for each model in the current data. Results yielded *BF* Model A = 3.99, *BF* Model B = 0.51, indicating that the data provide 3.99 times more support to Model A than the unconstrained (null) model, whereas only 0.51 times more support to Model B than the unconstrained (null) model. In addition, by dividing these Bayes factors, we found *BF* Modal A > Model B = 7.82, indicating that the data provide 7.82 times more support for Model A than Model B.

2 Again we conducted Bayesian analyses to examine the potential treat effect on the increase in happiness. Results yielded *BF* Model A = 7.39, *BF* Model B = 0.69, indicating that the data provide 7.39 times more support to Model A than the unconstrained (null) model, whereas only 0.69 times more support to Model B than the unconstrained (null) model. In addition, by dividing these Bayes factors, we found *BF* Modal A > Model B = 10.71, indicating that the data provide 10.71 times more support for Model A than Model B.

3 Again we conducted Bayesian analyses to examine the potential treat effect on the increase in happiness. Results yielded *BF* Model A = 9.19, *BF* Model B = 0.96, indicating that the data provide 9.19 times more support to Model A than the unconstrained (null) model, whereas only 0.96 times more support to the Model B than the unconstrained (null) model. In addition, by dividing these Bayes factors, we found *BF* Modal A > Model B = 9.57, indicating that the data provide 9.57 times more support the Model A than Model B.

4 https://www.hofstede-insights.com/product/compare-countries/
